# Experimental uncoupling of hosts and endosymbionts

**DOI:** 10.1128/mbio.01116-24

**Published:** 2024-07-19

**Authors:** Honglin Feng, Alex C. C. Wilson

**Affiliations:** 1 Department of Entomology, Louisiana State University AgCenter, Baton Rouge, Louisiana, USA; 2 Department of Biology, University of Miami, Coral Gables, Florida, USA; University of Hawaii at Manoa, Honolulu, Hawaii, USA; Emory University, Atlanta, Georgia, USA

**Keywords:** endosymbiosis, Fukatsuia, axenic culture

## Abstract

Many organisms harbor heritable bacterial symbionts that offer context-specific benefits to their hosts. In some of these symbioses, symbionts live inside host cells as endosymbionts. Studying the biology of endosymbiosis is challenging because it is hard to independently cultivate hosts and endosymbionts. A recent study, using a simple defined growth medium at ambient temperature, established an axenic culture of the pea aphid’s heritable bacterial endosymbiont, *Candidatus* Fukatsuia symbiotica (G. P. Maeda, M. K. Kelly, A. Sundar, and N. A. Moran, mBio 15:e03253-23, 2024, https://doi.org/10.1128/mbio.03253-23). Notably, the monoculture was capable of host recolonization, was stably transmitted, and returned similar host phenotypes to those observed in native infections. This advance in uncoupling the cultivation of an endosymbiont and its host opens avenues for genetic manipulation of the endosymbiont that will facilitate hypothesis-driven work to explore the mechanisms of host-endosymbiont biology and potentially facilitate the development of symbiont-mediated practical-application biotechnologies.

## COMMENTARY

Anyone who has spent time with a 4-year-old has likely experienced a conversation-driven forward by the child repeatedly asking “What?,” “How?,” or “Why?”. Progress addressing the most pressing outstanding questions in the study of endosymbiosis has been hampered by our ability to answer “What?,” “How?,” or “Why?” questions about the cellular and molecular processes that undergird the transmission and persistence of endosymbionts. The methodological advance made by Maeda et al. (1) in culturing *Candidatus* Fukatsuia symbiotica is significant because it opens the door to addressing fundamental questions, such as what genes underlie symbiont evasion of host immunity, how cellular homeostasis is maintained in host bacteriocytes, and why gene loss in symbiont genomes seems to follow such a predictable trajectory.

The first question that often arises in symbiotic research is what are the players in these intricate relationships? In natural settings, identifying these players is challenging because they exist as members of complex communities, and can be found in low abundance relative to other community members. For example, aphids not only carry *Ca*. F. symbiotica inconsistently but also confine it to specific tissues in extremely low abundance. Furthermore, *Ca*. F. symbiotica typically coexists with a myriad of symbiotic microbes, including the obligate endosymbiont, *Buchnera aphidicola*, and other facultative symbionts that can include *Hamiltonella defensa*, *Regiella insecticola*, *Rickettsiella viridis*, and *Serratia symbiotica* (2). Cultivating these symbionts in monoculture represents the first step toward untangling their identities from the natural complexity because it facilitates the generation of high-quality genomes and transcriptomic data to aid genome annotation (3, 4). Of course, such cultivation also poses limitations, for example, it would be difficult to extend transcriptomic and protemic studies beyond their application as facilitators of genome annotation because gene and protein expression can reasonably be expected to be different in axenic culture versus in endosymbiosis.

Another question in symbiosis research is how do endosymbionts evade host defenses and establish symbiotic relationships? While we cannot rewind the clock to witness these moments that occurred in the past, the inability of ancient endosymbionts to survive outside their hosts has made it challenging to understand this process. The axenic culture of *Ca*. F. symbiotica can readily re-establish infection in aphid hosts and persist across generations, enabling researchers to gain insights into host invasion dynamics, tissue tropism, and pathology (5, 6). Following the reintroduction of *Ca*. F. symbiotica to the aphid host, Maeda et al. meticulously tracked the symbiont’s invasion and transmission during aphid embryogenesis. That work returned a vivid visualization of the host invasion and tissue tropism process, sparking new hypotheses for further investigation. For example, we found it intriguing that *Ca*. F. symbiotica appeared to congregate at the tail end of *Buchnera* invasion (see Fig. 4B in reference 1), suggesting to us the possibility that *Ca*. F. symbiotica hijacks *Buchnera*’s invasion of the host, a phenomenon worthy of deeper exploration.

A third question concerns why gene loss in symbiont genomes seems to follow such a predictable trajectory. Among the many intriguing observations in the quest to culture endosymbionts is the remarkable correlation between culturability and genome size, where endosymbionts with larger genomes are more likely to be culturable (7). In addition to the pea aphid, *Ca*. F. symbiotica naturally infects various aphid species. In the aphid *Cinara confinis*, *Ca*. F. symbiotica is transitioning to a co-obligate status, supporting *Buchnera’s* decaying function (2). Culturing other strains of *Ca*. F. symbiotica with diverse genome sizes will facilitate experimental work aimed at understanding the trajectory of genome size reduction in bacteria that adopt an intracellular lifecycle, and help to shed light on the role of conflict in host/endosymbiont coevolution (8).

Most excitingly, the monoculture of *Ca*. F. symbiotica, independent of insect cells, and its reintroduction potential pave the way for the development of symbiont-mediated biotechnologies via genetic manipulation. One possibility is the development of a plasmid transformation protocol to enable the expression of foreign genes, such as reporter genes for *in vivo* tracking of endosymbionts or manipulation of host biology. Previously, the culture of a tsetse fly endosymbiont, *Sodalis glossinidius,* was engineered to express a single-domain antibody to interfere with trypanosome infection (9). Another type of genetic manipulation that is now a possibility involves making genetic modifications of the Fukatsuia chromosome to facilitate functional analyses. Previous work in *S. glossinidius* used mutagenesis to facilitate the identification of the type III secretion system crucial for tsetse fly cell invasion (10). Outside questions fundamental to understanding the biology of endosymbiosis, many insects, including aphids, pose significant agricultural threats. Thus, the technical advance of the axenic culture of an aphid symbiont by Maeda et al. also holds promise for the deployment of applied biotechnology in agricultural pest control. For instance, endosymbionts can be engineered to continuously express specific interfering double-stranded RNA constructs that target host genes in the development of symbiont-mediated RNA interference for pest control (11–13). Such modification of a symbiont genome without altering the host genome is commonly known as paratransgenesis, an approach that leverages the biology and evolution of natural symbionts, thereby facilitating the spread of the engineered symbiont through host populations to achieve effective control of disease vectors (14).

The generic name “Fukatsuia” of *Ca*. F. symbiotica was given in 2017 to honor Dr. Takema Fukatsu’s contributions to the identification of this secondary symbiont (15). Experimental work has demonstrated *Ca*. F. symbiotica to be a versatile symbiont of aphids, providing protection against heat, pathogens, and parasitoid wasps (16). The axenic mono-culture established by Maeda et al. will elevate *Ca*. F. symbiotica to a model of insect bacterial endosymbiosis, enabling researchers to address fundamental “What?,” “How?,” or “Why?” questions *in vivo*. Furthermore, this technical advance opens the door to the use of *Ca*. F. symbiotica as a platform for the development of symbiont-mediated biotechnologies, including paratransgenetic techniques for controlling aphid pests and plant disease transmission (14).
